# A polo ball in the right atrium, importance of echocardiographic characteristics of intracardiac myxomas: a case report

**DOI:** 10.1186/s13256-023-04130-6

**Published:** 2023-09-21

**Authors:** Mira Hamdan, Boutros Alam, Antoine Kossaify

**Affiliations:** Cardiology Division, Saint Esprit Kaslik University USEK, Hospital Notre Dame des Secours, Saint Charbel Street- PO Box # 3, Byblos, 12345 Lebanon

**Keywords:** Echocardiography, Myxoma, Right atrium, Trepopnea, Case report

## Abstract

**Background:**

Myxomas are the most prevalent type of heart tumors, mainly occurring in the left atrium, with approximately 20% of cases found in the right atrium. Timely diagnosis and appropriate management of myxomas are crucial for favorable outcomes and to minimize complications.

**Case presentation:**

A 77-year-old Asian male with no significant medical history presented with intermittent trepopnea and palpitations. Physical examination revealed regular heart sounds and no other relevant findings. A transthoracic echocardiogram showed a large, round-shaped, smooth-edged mass with diameter of 86 mm, occupying most of the right atrium. Mild tricuspid flow obstruction and mild left ventricular systolic dysfunction were also observed. Cardiac tomography confirmed the size, smooth edges, and showed microcalcifications of the mass, with no invasion of surrounding tissues. Surgical intervention successfully removed the spheroid mass, leading to the alleviation of symptoms. Histopathology confirmed the myxoma nature of the mass. A comprehensive discussion based on relevant medical literature is provided, with emphasis on echocardiographic characteristics of the mass with relation to potential embolic disease.

**Conclusion:**

This case shows an atypical presentation of an exceptionally large myxoma in the right atrium, resembling the size of a polo ball, in a patient presenting with supraventricular arrhythmia and trepopnea. Early diagnosis and appropriate management played a vital role in achieving a successful outcome for the patient.

## Introduction

Primary cardiac tumors are rare, with approximately 75% of them being benign. Among benign tumors of the heart, myxomas account for nearly 50% of cases; myxomas occur more frequently in females, and the average age of occurrence is 56 years [1]. In addition, cardiac myxomas are predominantly found in the atria, with over 80% in the left atrium and almost 20% in the right atrium [2].

The clinical presentation of cardiac myxomas varies depending on factors such as tumor size, texture, and location [3]. Typically, patients experience systemic symptoms such as fever, arthralgias, weight loss, and fatigue. Obstruction-related symptoms such as dyspnea, trepopnea, and syncope may also manifest, along with embolization manifestations [2]. In this report, we present a case of a huge myxoma in the right atrium, resembling the size of a polo ball. We include a comprehensive review of relevant literature and discussion, emphasizing the significance of echocardiographic characteristics in the management and prevention of complications associated with myxomas.

## Case presentation

**Fig. 1:**
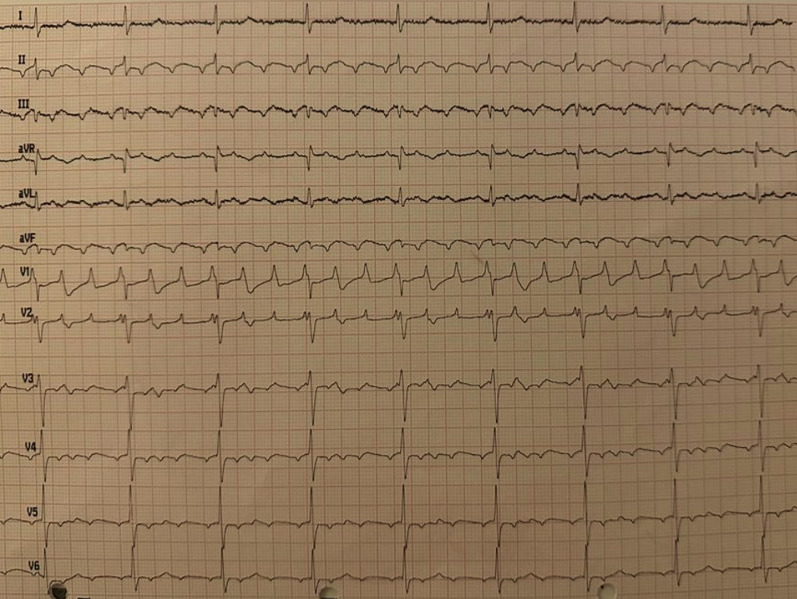
Preoperative EKG trace showing atrial tachycardia with ventricular response around 60 bpm

A 77-year-old Asian man with no significant medical history presented with mild and intermittent left trepopnea (shortness of breath while lying on the left side), and palpitations since nearly 2 months. He had no syncope or fainting episodes, also no fever, arthralgias, weight loss, or fatigue. His main risk factors were tobacco smoking, age, and a sedentary lifestyle. There was no relevant medical family history. He was not on any long-term medications, except occasional analgesics for headaches. Physical examination revealed regular heart sounds, with no abdominal distension or lower legs edema. EKG showed atrial tachycardia with an average ventricular rate of 60 bpm (Fig. 1). Chest X-rays showed normal lung parenchyma, with enlarged right lower curve suggestive of right atrial enlargement. Laboratory results (C-reactive protein, complete blood count, hemoglobin level (13 g/dL), and serum interleukin-6 level (7 pg/mL)) were normal.

**Fig. 2:**
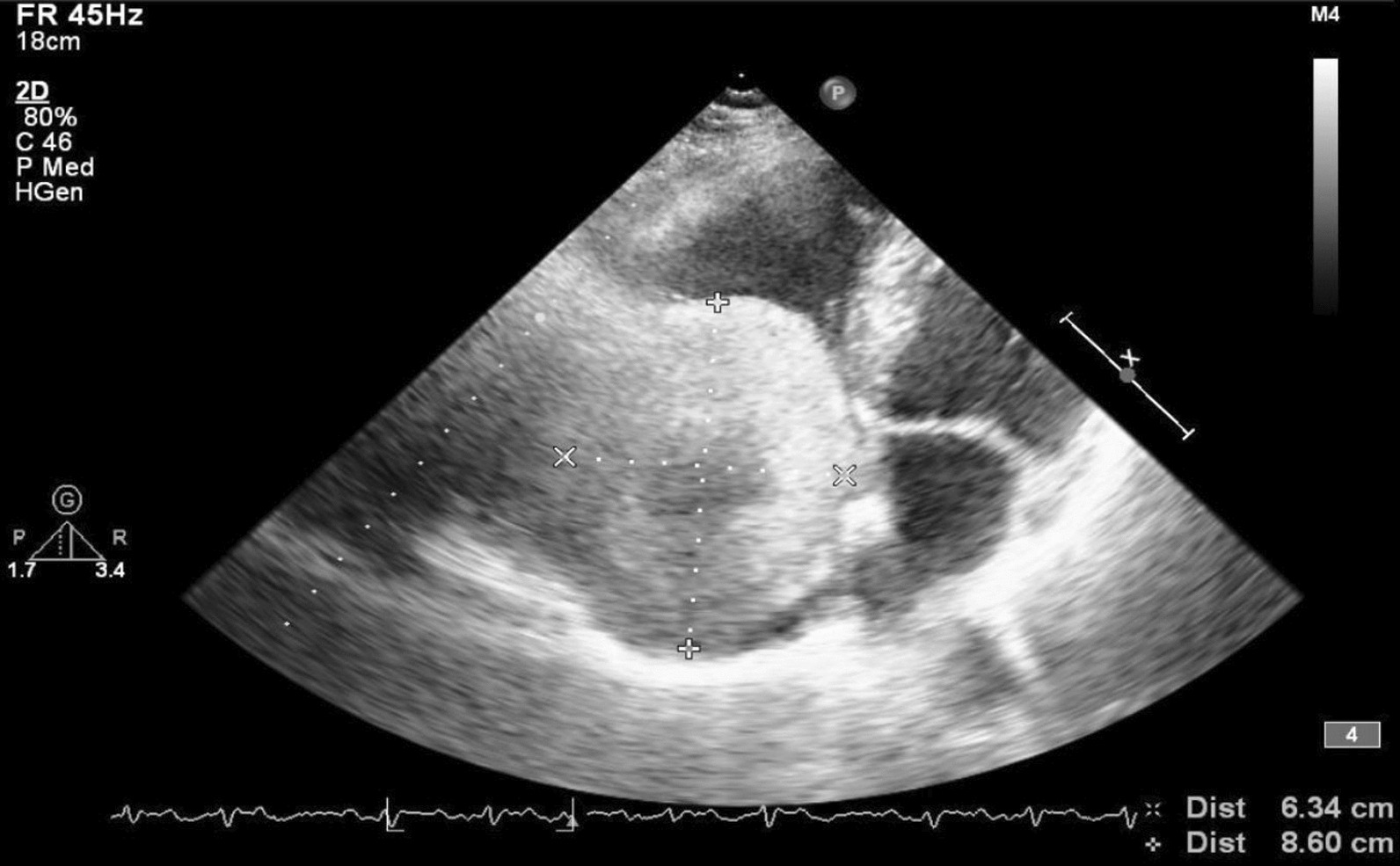
Modified apical four cavities view showing a huge intraatrial mass of 63 × 86 mm, with mechanical pressure on tricuspid valve and mild intrusion into the right ventricle

**Fig. 3:**
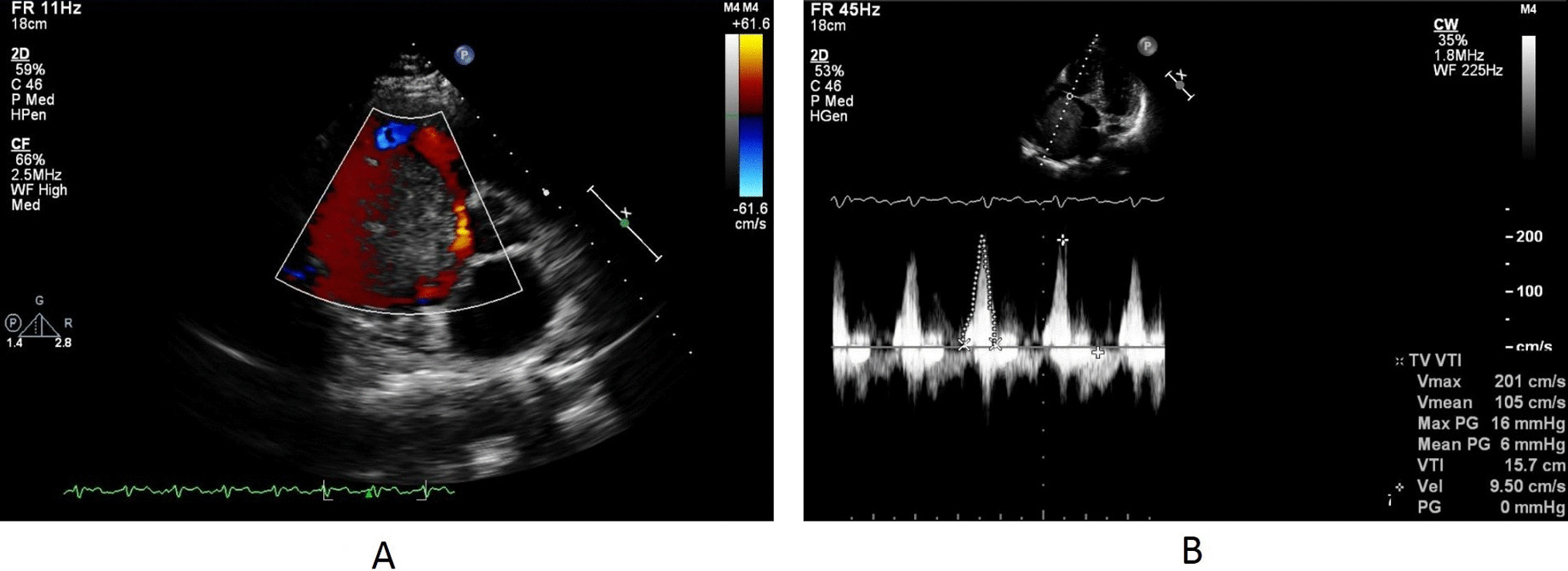
**A** PSAX view showing the blood flow persisting on the periphery of the mass. **B** Apical four cavities view with spectral Doppler on tricuspid valve showing a mean gradient of 6 mmHg across the valve and designating a mild to moderate obstruction syndrome

**Fig. 4:**
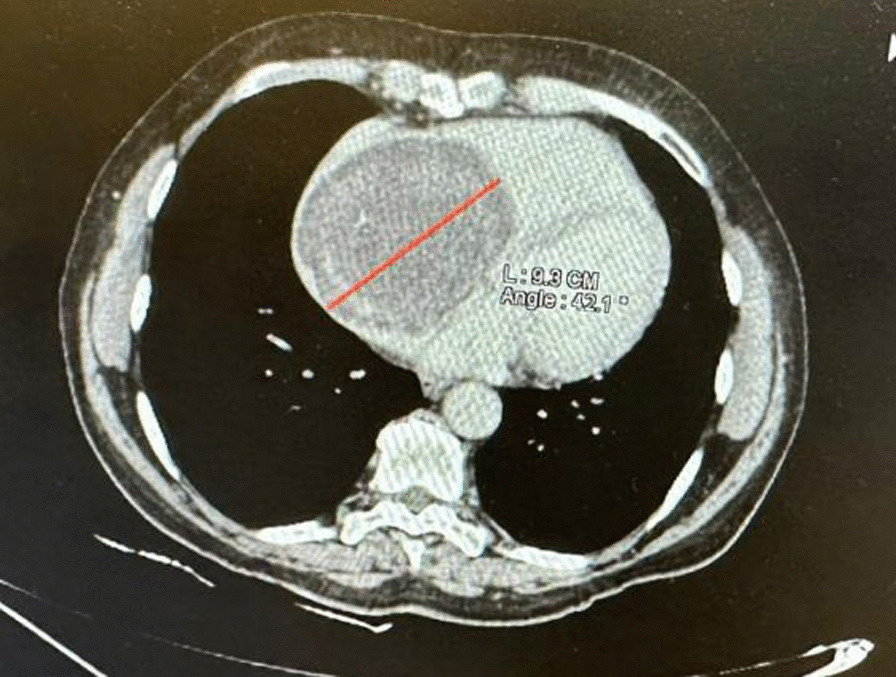
Cardiac tomography showing a round-shaped mass with maximal diameter of 9.3 cm located in the right atrium, with mild calcification and without invasion of the surrounding tissues

Transthoracic echocardiography (TTE) using a Philips iE33 ultrasound system revealed a large spheroid mass in the right atrium, measuring 86 × 63 mm, with a smooth edge. The mass occupied most of the right atrium; there was mild functional tricuspid flow obstruction, slightly reduced left ventricular systolic function, and normal inferior vena cava size. There were difficulties in assessing pulmonary artery pressure due to the patient’s dyspnea while lying on the left side (left trepopnea) and limited sonographic signal (Fig. 2). No stalk was observed, and three-dimensional echo imaging was unavailable. Figure 3A shows blood flow around the mass, while Fig. 3B reveals mild functional tricuspid functional stenosis caused by the obstructive effect of the mass; image acquirement while in dorsal supine or right decubitus positions to evaluate flow changes was difficult owing to poor sonographic signal. The patient declined transesophageal echocardiography. Cardiac tomography revealed a mass in the right atrium with a 9.3 cm diameter, smooth edges, along with microcalcifications and without invasion of the surrounding tissues (Fig. 4). Coronary angiogram results indicated normal coronary arteries.

**Fig. 5:**
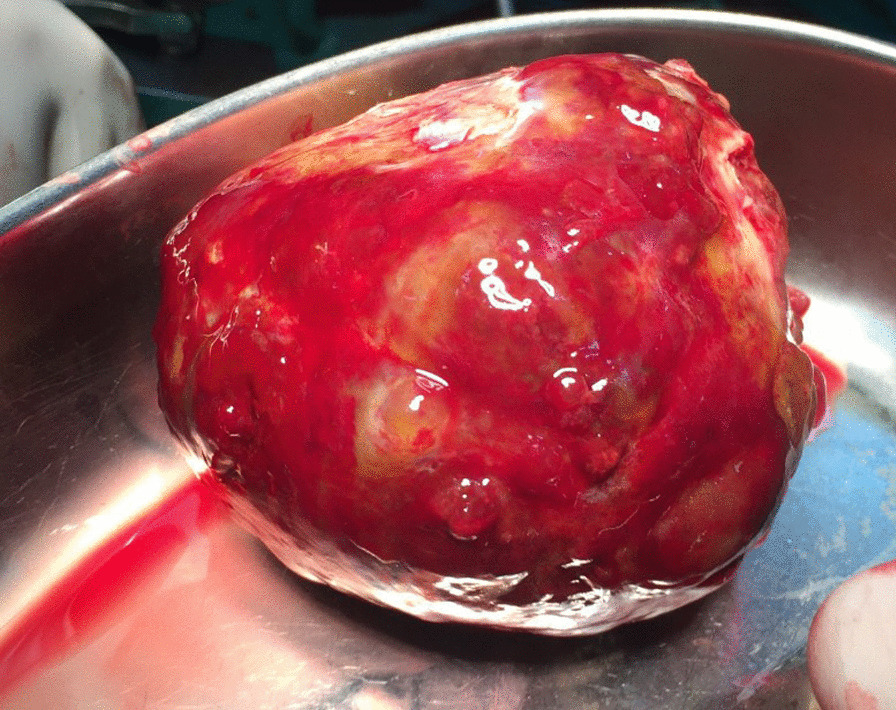
The resected mass had a spheroid shape with smooth edges; a short and small pedicle (not visible in the image) was attached to the right atrial free wall

After discussion with the patient, surgical removal of the mass was performed via median sternotomy. The resected mass had a short and small pedicle attached to the right atrial free wall; it had a spheroid shape with smooth edges, measuring approximately 8.5 cm in diameter (Fig. 5). No tissue invasion was observed. Histologic analysis confirmed the myxoma nature of the mass, with lipidic cells and scant eosinophilic cytoplasm embedded in a vascular myxoid stroma. There was minimal calcification and central tumor necrosis, with absence of interleukin-6 expression on immunohistochemical study. Following the surgery, the patient became asymptomatic with restored normal sinus rhythm. He was discharged a few days later.

## Discussion

This report highlights an exceptional case of a large right atrial myxoma resembling a polo ball. Despite its size, the patient had few symptoms. Typically, cardiac myxoma symptoms arise from flow obstruction, embolization, and the production of interleukin-6 by tumor cells; in addition, symptoms vary based on tumor size, location, and texture. Interleukin-6 release causes systemic symptoms or paraneoplastic syndrome such as fever, weight loss, and arthralgia [4, 5]. Furthermore, symptoms may be position-specific, such as trepopnea, owing to mass motion hindering blood flow. 

Cardiac presentation with supraventricular arrhythmia is rare and can be attributed to mechanical irritation and wall stretch of the atrium caused by the intracavitary mass [6, 7]. Trepopnea in the left decubitus position is believed to be linked to mass mobility and partial tricuspid flow obstruction. Interestingly, despite the large mass, blood flow persisted along the peripheral edge of the huge myxoma [7] (Fig. 3B).

The absence of interleukin-6 expression explains the lack of systemic symptoms in this patient, as approximately 24% of cardiac myxomas do not show interleukin-6 expression [8]. The absence of embolic disease as clinically estimated (given that no pulmonary imaging was performed in this regard) is explained by the smooth contour without villous or polypoid edges, and this phenomenon is already reported [9, 10].

The presence of supraventricular arrhythmia, irregular tumor surface, increased tumor size, and dilated atria are linked to an increased risk of embolism [11]. Additionally, tumors displaying high mobility are predictive of embolic events [12]. TTE is usually adequate for the diagnosis, showing tumor location and size, while attachment and mobility can also be assessed. Atrial myxoma must be differentiated from atrial thrombus, which usually has a layered appearance; also, the presence of a stalk and mobility favors atrial myxoma. Doppler echocardiography can show the hemodynamic consequences of atrial myxoma [1], as seen in this patient (Fig. 3C). Transesophageal echocardiography and three-dimensional (3D) echocardiography offer better visualization of the implantation site. Second-level diagnostic procedures (cardiac computed tomography, photon emission tomography (PET), cardiac magnetic resonance) are useful for better mass profiling and tissue characterization; also, PET helps in the diagnosis of metastatic masses as well as bacterial vegetations [1].

Right atrial myxomas can cause symptoms of right-sided dysfunction, such as peripheral edema, and ascites, but these symptoms were absent in this patient [13]. Clinical approach and imaging are crucial for accurate diagnosis, differential diagnosis, and for providing essential data for the surgeon. It is important to recognize normal anatomic variants that should not be mistaken for pathological entities. Intracavitary cardiac masses can be either benign or malignant. Benign masses include myxoma, cardiac papillary fibroelastoma, rhabdomyoma, lipoma, and hemangioma, while common malignant masses include angiosarcoma and metastases. Nontumoral masses consist of cysts, mitral caseous degenerative formations, thrombi, and vegetations [1]. Importantly, patients with Carney complex, an autosomal dominant multiple neoplasia syndrome, have high recurrence rate of myxoma (up to 40%) [14]. Surgical removal via median sternotomy is the conventional approach to treat cardiac myxoma; minithoracotomy with robotically assisted surgery has been reported as a safe and feasible method, resulting in shorter hospital stays [15].

## Study limitations

Transesophageal echocardiography was not performed as the patient declined the examination. However, it is worth noting that more accurate sonographic data could have been acquired if the procedure had been conducted. This additional data might have included information about the presence of stalk, surface echotexture, and a better definition of the mass anatomy, among other relevant details.

## Conclusion

Accurate identification and characterization of cardiac masses are crucial owing to the potential for serious complications. In this case, a large myxoma was diagnosed in the right atrium, and despite its significant size, the patient exhibited minimal symptoms. The atypical presentation as atrial tachycardia and trepopnea can be attributed to stretching of the right atrial wall and the mobility of the myxoma, causing intermittent partial flow obstruction. TTE is presented as the preferred diagnostic tool for initial diagnosis and characterization of cardiac myxomas, with emphasis on echocardiographic characteristics of the mass with relation to potential embolic disease.

## Data Availability

Not applicable.
